# Remotely delivered cognitive therapy for social anxiety disorder in adolescence: Preliminary efficacy evidence based on changes throughout treatment

**DOI:** 10.3389/fpsyg.2022.915677

**Published:** 2023-01-10

**Authors:** Paula Vagos, Diana Vieira Figueiredo, Ana Ganho-Ávila, Andreas Mayr, Daniel Rijo

**Affiliations:** ^1^Instituto de Desenvolvimento Humano Portucalense, Universidade Portucalense Infante D. Henrique, Porto, Portugal; ^2^Center for Research in Neuropsychology and Cognitive and Behavioral Intervention (CINEICC), Faculty of Psychology and Educational Sciences, University of Coimbra, Coimbra, Portugal; ^3^Department of Medical Biometry, Informatics and Epidemiology, University Hospital Bonn, Bonn, Germany

**Keywords:** cognitive therapy, social anxiety disorder, adolescence, change throughout therapy, multi-informant

## Abstract

Cognitive therapy has been established as the frontline treatment for adults with social anxiety disorder (SAD); its efficacy with socially anxious adolescents is incipient but promising. This work investigated change in social anxiety symptoms reported by adolescents and their therapist as they go through remotely delivered 10-session cognitive therapy (i.e., CT@TeenSAD). Participants were 21 adolescents (81% females; *M*_age_ = 16.10) diagnosed with SAD. They reported on change on their social anxiety symptoms at the beginning of each session; their therapist reported on how their symptoms had improved at the end of each session. Results, though preliminary, show that sessions had a significant impact on self- and therapist reported change, with consistent and continuous improvement across intervention sessions. Gender did not impact on that change, but therapist did: though the same pattern of change emerged for both therapists, it was more evident for the therapist with the greatest previous clinical experience. Overall, current findings align with the cognitive therapy framework of progressive gains throughout therapy. They also add evidence on the applicability and usefulness of an online cognitive approach to adolescents diagnosed with SAD.

## 1. Introduction

Social anxiety disorder (SAD) refers to the marked and persistent fear of social and/or performance situations in which one may be exposed to the scrutiny of others and of that scrutiny resulting in criticism, humiliation, or social rejection. This fear may be generalized to interaction, observation and performance social events or it may be specific to performance social events only (American Psychiatric Association, 2013). SAD typically arises during adolescence (Stein et al., 2017), with point-estimated prevalence rates ranging between 1.29% (Jystad et al., 2021) to 5.7% (Aune et al., 2022). Though SAD is more prevalent in females than in males (e.g., Aune et al., 2022), its clinical presentation and associated impairment does not differ by gender (Dahl and Dahl, 2010). If left untreated, SAD tends to maintain a chronic and unremitting course (Stein et al., 2017) and has frequent comorbidity with other anxiety and mood disorders (Garcia-Lopez et al., 2016; Jystad et al., 2021). The presence of SAD in adolescence is associated with overall severe functional impairments (Stein et al., 2017), including decreased quality in interaction with potential romantic relationships (Hebert et al., 2013) and with friends (Van Zalk et al., 2011), and decreased academic achievement (Vilaplana-Pérez et al., 2021). Given that social interactions are a vital aspect of adolescents’ developmental experiences and goals (Orben et al., 2020), it seems paramount to explore psychotherapeutic ways of effectively changing the course of SAD in adolescence.

Clark and Wells’ model (1995) offers a strong and evidence-based theoretical background for the understanding of SAD. Though developed to explain the maintenance of adult SAD, it has been proven applicable to adolescents diagnosed with SAD (Leigh and Clark, 2018a,b). According to this model, social situations are seen as potentially threatening, triggering a chain of self-perpetuating cognitive, affective, physiological, and behavioral responses that maintain individuals’ distress. These responses include several inter-linked processes such as excessive self-focused attention, decreased processing of external cues, use of interoceptive information to infer how one appears to others, use of safety behaviors, misinterpretation of somatic and cognitive symptoms and pre- and post- event processing (Clark and Wells, 1995; Clark, 2001; Leigh and Clark, 2018a,b). These processes have been proven relevant for understanding adolescents’ social anxiety. For example, biased social cognitions were found to predict safety-behaviors (Schreiber et al., 2012) and social anxiety (Vieira Figueiredo et al., 2021). The role of pre- and post-event processing (Schreiber et al., 2012) and of self-focused attention on social anxiety have also received evidence, though the later has been proposed to anticipate both cognitions (Schreiber et al., 2012) and safety behaviors (Vieira Figueiredo et al., 2021). Moreover, cognitive therapy for SAD (CT-SAD; based on Clark and Wells’ cognitive model of social anxiety), has been found to be highly efficacious (Mayo-Wilson et al., 2014) and cost-effective for adults (Mavranezouli et al., 2015) and to be promising for adolescents with SAD (i.e., symptom remission and associated gains in comorbid symptom, as well as reduction in social anxiety related thoughts, beliefs and safety behaviors), when implemented by specialized (Leigh and Clark, 2016) and non-specialized (Leigh et al., 2021) therapists.

More recently, Leigh and Clark (2022) found internet-delivered CT-SAD for adolescents to outperform a waitlist condition concerning several symptoms associated with social anxiety and in relation to remission of SAD diagnosis for 77% of participants at postintervention. This form of delivered is based on an online platform that adolescents use to go through core intervention models. In addition, each participant has weekly phone-calls with therapists and goes through a self-focused attention and safety behavior experiment with video feedback using videoconference. Resorting to videoconference as a means of delivering CT has been suggested as relevant and useful, as long as some cautions are considered (e.g., assuring the adolescent has access to a private connection and space to participate in the intervention and having proficiency in the use of the videoconferencing platform used to deliver the intervention; Warnock-Parkes et al., 2020). Nevertheless, the efficacy of full synchronous online CT for SAD in adolescence (i.e., conducting sessions using live videoconference) has not been investigated so far. Online synchronous psychotherapy may be particularly relevant to SAD: because it is more accessible it may help circumvent the typically low rates of help-seeking behavior of adolescents with SAD symptoms (McDonagh et al., 2022). The present work aimed to explore preliminary efficacy data regarding self and therapists’ reports on the change of social anxiety symptoms across an online 10-session manualized CT program (CT@TeenSAD) applied to adolescents with generalized SAD. Based on previous promising findings for the CT-SAD for adolescents (Leigh and Clark, 2016; Leigh et al., 2021) and on findings that point to social anxiety symptoms progressively diminishing during the course of cognitive-behavioral treatment (Zaider et al., 2003; Hayes et al., 2008) and of internet-delivered CT (Leigh and Clark, 2022), we expected both self and therapists’ ratings of social anxiety symptoms to decrease as intervention progresses (see Intervention section below). As secondary goals, we aimed to explore the effect that other relevant variables may have on change throughout the intervention, namely gender and therapist. Because male and female adolescents’ social fears seem to present similarly despite diverse prevalence rates (Dahl and Dahl, 2010), we expect sex to have no significant effect on change throughout the CT@TeenSAD program. Also, because CT has shown promise with therapists of diverse proficiency in that specific therapy (Leigh and Clark, 2016; Leigh et al., 2021), we also expect therapist to have a non-significant effect on change throughout the CT@TeenSAD program.

## 2. Materials and methods

This work is part of the ongoing research project *TeenSAD: Changing the Course of Social Anxiety in Adolescence* (ClinicalTrials.gov Identifier: NCT04979676).^1^ This research project was approved by the Ethics Committee of the host institution prior to any recruitment or data collection procedure taking place. This research proposes to investigate the efficacy of cognitive therapy, compassion focused therapy, and acceptance and commitment therapy on adolescent SAD, against a waitlist control condition. As such, *a priori* sample size determined using G*Power (four conditions across four assessment moments) was set at a minimum of 72 participants (i.e., 18 per group), with power analysis placed at 0.95, an expected effect size of 0.25 and an expected correlation between repeated measures placed at 0.30. Only the CT group will be considered in the current work.

### 2.1. Recruitment procedures

The goals and procedures of this research were presented to several schools located in the center and north regions of Portugal, 14 of which became partners in the recruitment procedures. Specifically, they shared the study with their 10th and 11th grade students and their legal guardians and requested verbal assent and written informed consent, respectively, for students to take part on the screening phase of recruitment. This phase consisted of students filling in the Portuguese version of the Social Anxiety Scale for Adolescents (Cunha et al., 2004). Students scoring one standard deviation above the mean found for a large adolescent normative sample were then called for the assessment phase. The assessment phase consisted of individual assessment *via* a structured clinical interview (i.e., the Portuguese version of the Mini International Neuropsychiatric Interview for Children and Adolescents; Rijo et al., 2016), which was used for defining the presence of inclusion criteria (i.e., primary diagnosis of generalized SAD) and absence of exclusion criteria (i.e., primary diagnosis other than SAD, psychotic symptoms, suicidal risk, special education needs, and receiving any form of psychological support at the moment of recruitment). Participants fulfilling these criteria and their legal guardians were asked for additional consent to be assigned to an experimental condition. Assignment was randomly made by the responsible investigator, who was blind to any information on the participant except that they had fulfilled all inclusion and exclusion criteria. Specifically, that investigator would blindly and manually take one of the available options (i.e., control vs. experimental) for each selected participant until the pre-defined sample size for each group was obtained.

### 2.2. The CT@TeenSAD intervention

The CT@TeenSAD was developed based on the intervention manual for CT for SAD in adolescence by Leigh and Clark (2018a,b). It consists of 10 individual weekly sessions; two booster sessions are delivered monthly after the core program has ended but will not be considered in the current work. Sessions 2 through 10 have a similar structure that includes three parts: welcoming (i.e., short overview of insights brought about by the previous session, and interactive discussion of the adolescents’ social experiences between sessions), theme development (i.e., one or more exercises – including a specific technique and insight into its usefulness – designed to explore the theme of the sessions), and conclusion (i.e., collaborative and interactive synthesis of the sessions’ core aspects). Session 1 differs only in that the welcoming has to do with defining rules and goals for the intervention process. Although the research project had intended to deliver the intervention face-to-face, the COVID-19 pandemic led to adapting the intervention to be delivered remotely *via* videoconference using the Pro version of Zoom (Zoom Video Communications, Inc., 2020). This happened before any intervention case had begun and so all sessions were delivered remotely to all participants. The guidelines provided by Warnock-Parkes et al. (2020) to implement remotely delivered cognitive therapy were followed (e.g., having the camera on during the sessions and self-view hid to mimic real life interactions, use of screen sharing to mimic collaborative work, assurance of technical conditions and of a private and quiet location where sessions could occur).

The 10 sessions are organized into four sequential modules. The first module (sessions 1–3) focuses on deriving an individualized cognitive model of social anxiety and on discovering the impact of self-focused attention and safety behaviors on social anxiety. The second module (sessions 4–5) addresses training attention flexibility and planning for more accurate pre- and post-event processing. The third module (sessions 6–9) prompts participants to perform in-session behavioral experiments during interactions and performance in social events, using video-feedback for its analysis, and practicing behavioral experiments between sessions. The fourth module (session 10) reviews gains/goals attainment and addresses relapse prevention. See more detailed information at Supplementary Table S1.

Interventions lasted from February to December 2021. Therapists were one doctor in psychology (Therapist A) and one master in psychology (Therapist B) who had similar experience in this particular intervention approach, though the fist had more clinical experience overall. Both were involved in developing the intervention manual and had weekly inter and supervision meetings with the responsible investigator of this research project to ensure comprehension of the sessions to be applied and continued treatment fidelity. After each session, therapists filled in a form about adherence to the manual. These forms were assessed by the responsible investigator; no significant deviations to the manual were reported.

### 2.3. Data analyses

As per our primary goal, linear mixed-effect models were used to investigate the longitudinal effect of session on self-reported (i.e., SASCI) and on therapist-reported (i.e., CGI-SA) change since the beginning treatment. Session was coded as 2 = session 2 until 10 = session 10 and was taken as the fixed effect, as was severity rated from 1 = normal symptomatology to 7 = very severe symptomatology by the therapist at diagnostic assessment, to adjust for possible confounders. Additionally, participant-specific random intercepts were incorporated to account for the nested data structure. The further explore the longitudinal trajectory of SASCI and CGI-SA, the session effect was also analyzed *via* planned pairwise comparisons using the Bonferroni correction on eight comparisons. Specifically, we compared ratings taken from session 2 – which was the first-time ratings were taken – with ratings taken from all other sessions. This option was based on acknowledging the constructs assessed by the outcome instruments, which ask participants to rate change in each session compared to a baseline moment (see instrument description below). As per our secondary goals, subsequent linear mixed-effects models were computed wherein participants’ sex (1 = female, 2 = male) and therapist (1 = Therapist A, 2 = Therapist B) were added separately to the previous models as fixed effects. There was no missing data. All analyses were performed *via* the statistical programming environment R (R Core Team, 2017) using the lme4 and the asbio add-on packages; two-tailed *α* < 0.05 was applied to all analyses. Model formulas for all linear-mixed effects models conducted with this research can be found in Supplementary Table S2.

### 2.4. Change outcome measures

The European Portuguese version of items for both instruments was achieved *via* translation and back translation processes made by members of the research team who are experts in SAD assessment and treatment.

#### 2.4.1. Social anxiety session change index

The Social Anxiety Session Change Index (SASCI; Hayes et al., 2008) consists of four items used to evaluate the perception of owns’ change in social anxiety symptoms since the beginning of treatment by individuals attending therapy for SAD (i.e., level of anxiety in social/performance situations, avoidance of those situations, concerns about being embarrassed or humiliated, and social anxiety-related daily interference). Items are scored using a 7-point scale ranging from 1 (much less than at the start of treatment) to 7 (much more than at the start of treatment). The SASCI has shown adequate internal consistency (*α* ≥ 0.84), validity in relation to self-reported change in fear of negative evaluation and in relation to therapist-reported social anxiety, severity of symptoms and improvement (Hayes et al., 2008). Because we wanted responses to reflect on the adolescents’ experience outside therapy (i.e., ecological change), items were presented to participants at the beginning of sessions 2 through 10. Cronbach’s alpha values for the aggregate of four items for each session varied between *α* = 0.76 for session 4 and *α* = 0.87 for session 5, except session 3 which had *α* = 0.55.

#### 2.4.2. Clinical Global Improvement - Social Anxiety

The Clinical Global Improvement - Social Anxiety (CGI-SA; Zaider et al., 2003) considers therapists’ perception of their patients presenting with SAD on overall severity of symptoms within the past week and on global improvement in comparison to a baseline moment. The therapist does ratings by selecting one of seven categories referring specifically to social anxiety-related symptoms and impairment (i.e., 1 = normal symptomatology to 7 = very severe symptomatology for the overall severity measure and 1 = very much worse to 7 = very much improved for the global improvement measure). The CGI-SA has shown construct validity in relation to patient-reported and other clinician administered measures of social anxiety and impairment (Zaider et al., 2003). The severity measure was used as indicative of pre-treatment severity and entered as a main effect to adjust for possible confounders to our findings (see Data analysis section). The change measure was taken as the dependent variable representing change across sessions. The recommendation is that this measure is filled in after close contact with the patient (Zaider et al., 2003). Accordingly, the severity of symptoms was rated immediately after the individual diagnostic interview and the global improvement measure was rated at the end of each session – starting on session 2 – so therapists could rely on the information provided by the adolescents on their experience outside therapy during the previous week. Means and standard deviations for therapist-reported change across intervention sessions can be found in Supplementary Table S3.

## 3. Results

### 3.1. Participants

**Figure 1:**
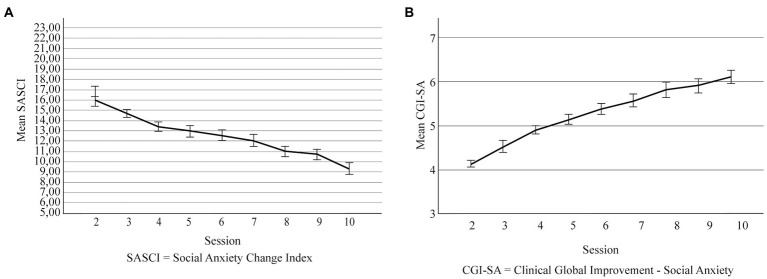
**(A)** Self-reported change across intervention sessions. **(B)** Therapist-reported change across intervention sessions. Error bars are ±1 SD. SASCI, Social Anxiety Session Change Index; CGI-SA, Clinical Global Improvement - Social Anxiety.

An initial pool of 1,495 students were screened, of which 388 were eligible for individual diagnostic assessment. Of those, 209 were interviewed by one of four masters in psychology who received specific training to apply the interview; 140 met all inclusion criteria and 92 were available to receive psychotherapy within this research project. Of those, 29 were assigned to CT@TeenSAD by the responsible investigator, using a randomized and parallel clinical trial methodology. Twenty-one participants were completers (Figure 1), including 17 female (81%) and 4 male participants (19%), aged between 15 and 17 at time of assignment (*M* = 16.10, SD = 0.77). Alternatively, two participants refused to start treatment and six participants ended treatment after 3–8 interventions sessions, mostly due to having achieved the desired change as early as session 5 (*n* = 3). Data from these participants was not considered in the current work as we intended to analyze the efficacy of the complete 10-session intervention.

Completers were attending the 10th (*n* = 8, 38.1%), the 11th (*n* = 11, 52.4%) or the 12th grade (*n* = 2, 9.5%) and came from a low (*n* = 6, 28.6%), medium (*n* = 12, 57.1%) or high (*n* = 3, 14.3%) socioeconomic household. All received a primary diagnosis of generalized SAD within the recruitment procedures of the current work; additionally, two participants had a diagnosis of generalized anxiety disorder, and one participant had a diagnosis of hyperactivity and attention deficit disorder. A minority of participants had previously received psychological support (*n* = 5, 23.8%), though only one had been for SAD symptoms. That support had ended on average 4.86 months ago (SD = 12.24).

### 3.2. Change outcomes throughout the CT@SAD intervention

#### 3.2.1. Self-reported change

**Figure 2:**
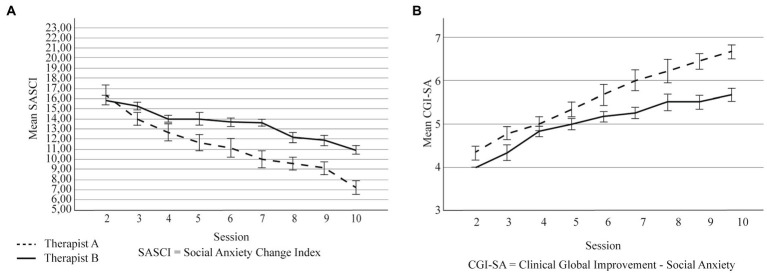
**(A)** Self-reported change across intervention sessions, by therapist. **(B)** Therapist-reported change across intervention sessions, by therapist. Error bars are ±1 SD. SASCI, Social Anxiety Session Change Index; CGI-SA, Clinical Global Improvement - Social Anxiety.

The overall model explained 74% of the SASCI change scores across sessions and the main effect of session was statistically significant for self-reported change (see Table 1). The ICC value of a random intercept model revealed that 27% of the variability of the SASCI was attributable to differences between patients. Pairwise comparisons additionally show that self-reported symptoms were similar between sessions 2 and 3, and then significantly decreased throughout the remaining sessions, in comparison with session 2 (*p* < 0.001; Figure 1A); see Supplementary Tables S3, S4 for descriptive values for the SASCI in each session and for between-session comparisons, respectively. As for our secondary goals, therapist (in addition to session) had a statistically significant main effect on self-reported change explaining 74% of the variance of the SASCI scores (see Table 2). Though self-reported symptoms continuously decrease throughout sessions for both therapists, this trend is more evident for therapist A (see Figure 2A). The main effect of participants’ sex (in addition to session) was not statistically significant (see Supplementary Table S5).

**Table 1:** Linear mixed model for session as fixed-effect explaining self and therapist-reported symptom change while adjusting for baseline severity.

Predictors	SASCI				CGI-SA			
		Estimates	*CI*	*p*	*df*	Estimates	*CI*	*p*	*df*
	(Intercept)	17.40	10.59–24.21	<0.001	184	4.02	2.43–5.61	<0.001	184
	Session	−0.74	−0.83 to −0.66	<0.001	184	0.24	0.21–0.26	<0.001	184
	Baseline severity	−0.24	−1.67 to 1.19	0.739	184	0.02	−0.32−0.35	0.929	184
Random effects									
	*σ^2^*	2.41				0.23			
	*τ_00_*	3.05_participant_				0.23_participant_			
	*ICC* for complete model/ *ICC* for random intercept only	0.56/0.27				0.40/0.13			
	*N*	21_participant_				21_participant_			
Marginal R2/ Conditional R2		0.405/0.738				0.494/0.697			

SASCI, self-reported symptom change; CGI-SA, therapist-reported symptom change. Observations = 189.

**Table 2:** Linear mixed model for session and therapist as fixed-effects explaining self and therapist-reported symptom change while adjusting for baseline severity.

Predictors	SASCI				CGI-SA			
		Estimates	*CI*	*p*	*df*	Estimates	*CI*	*p*	*df*
	(Intercept)	17.97	12.50–23.44	<0.001	183	3.87	2.74–4.99	<0.001	183
	Session	−0.74	−0.83 to −0.66	<0.001	183	0.24	0.21–0.26	<0.001	183
	Baseline severity	0.33	−0.87 to 1.52	0.589	183	−0.14	0.035–0.10	0.256	183
	Therapist	−2.28	−3.60 to −0.96	0.001	183	0.63	0.35–0.89	<0.001	183
Random effects									
	*σ^2^*	2.41				0.23			
	*τ_00_*	1.86_participant_				0.06_participant_			
	*ICC* for complete model/ *ICC* for random intercept only	0.44/0.27				0.22/0.13			
	*N*	21_participant_				21_participant_			
Marginal R2/ Conditional R2		0.535/0.737				0.612/0.696			

SASCI, self-reported symptom change; CGI-SA, therapist-reported symptom change. Observations = 189.

#### 3.2.2. Therapist-reported change

The overall model explained 69% of the variance of the therapist-reported change throughout sessions and session had a statistically significant main effect on that report (see Table 1). The ICC value of a model containing only the random intercept revealed that 13% of the variability of the CGI-SA was attributable to differences between patients. Pairwise comparisons between sessions show similar scores between sessions 2 and 3, and then a statistically and continuous improvement throughout the remaining sessions, in comparison with session 2 (*p* < 0.001; Figure 1B); see Supplementary Tables S2, S6 for on descriptive values for the CGI-SA in each session and for between-session comparisons, respectively. Therapist (in addition to session) also had a significant main effect on therapist-reported change, explaining 69% of those scores. Although both therapists report continuous improvement, that ascend is more pronounced for Therapist A (see Figure 2B). The main effect of participants’ sex (in addition to session) was not statistically significant (see Supplementary Table S5).

## 4. Discussion

The current work showed preliminary efficacy of synchronous and remotely CT delivered to adolescents with SAD; this online synchronous approach had been preliminarily shown feasible and useful using a case study approach (Ganho-Ávila et al., 2022). Specifically, we found preliminary evidence that both adolescents and therapists perceived a continuous decrease/improvement in social anxiety symptoms throughout the course of 10-intervention sessions. Change was also found for both therapists, though the most experienced therapist was associated with greater gains. This aligns with previous findings that change may come from therapists of diverse proficiency therapy (Leigh and Clark, 2016; Leigh et al., 2021); gains associated with those therapists had not been compared before. In our case, though both therapists had similar knowledge concerning CT, they had diverse levels of clinical experience in different models within a CBT approach. It may be the case that clinical experience allowed one therapist to greatly develop skills that are relevant and transversal (e.g., active listening, establishing of rapport) and so were also beneficial in the case of the CT@TeenSAD. Alike previous findings reviewed by Mululo et al. (2012), change paths were found to be similar across male and female participants, given that biological sex did not significantly impact change. Taken together, these findings strengthen the practical relevance of the CT@TeenSAD and the further study of its efficacy, as well as support the idea that gender-based differences for social anxiety may be quantitative (e.g., Asher et al., 2017) rather than qualitative, with CT being equally applicable to adolescent males and females.

Furthermore, findings generally concur with previous evidence on the applicability of CT to adolescents (Leigh and Clark, 2018a,b) and of its efficacy indicators (Leigh and Clark, 2016; Leigh et al., 2021). The continuous change throughout treatment for SAD had been advanced before for adults receiving cognitive-behavioral individual therapy (Goldin et al., 2014) and for adolescents receiving internet-delivered CT (Leigh and Clark, 2022). Current findings also add evidence to the progressive and wholistic nature of CT, in which previously learnt skills (e.g., flexibility in attention allocation) may support successful experiences later in therapy (e.g., behavioral experiments). Previous evidence had been found specifically on the relevance of dropping safety behaviors and self-focused attention (McManus et al., 2009) and of using video-feedback to get to a more realistic perspective on ones’ social performance (Warnock-Parkes et al., 2017). CT@TeenSAD considers both of those aspects in sessions 3–4 and in sessions 6–9 respectively, and so current findings add evidence to the importance of those intervention components in gradually reducing social anxiety symptoms.

Also in support of CT producing noteworthy and relevant changes in social anxiety symptoms is the fact that descriptive values of the SASCI and the CGI-SA found for the current sample at the 10^th^ session are lower than those found after 10 intervention sessions for self-reported change (Hayes et al., 2008) and after 12 intervention sessions for clinician-rated improvement (Zaider et al., 2003). In both cases, adults with SAD were receiving cognitive-behavioral therapy. Current findings also align with evidence on multi-informant change following a cognitive-behavioral approach to SAD in adolescence (Fox and Warner, 2017); because in our case therapists were delivering the intervention sessions (and were not independent observers as in Fox and Warner, 2017), therapist ratings may be based on more comprehensive information, resulting in more aligned perspectives between the adolescent and their therapist.

Given that, to our knowledge, the CT@TeenSAD intervention is the first to rely on video conferencing for adolescent SAD for the complete implementation of the intervention, current findings also add evidence to the previous assumption of the applicability and utility of remote CT (Warnock-Parkes et al., 2020), so long as specific aspects are carefully considered (e.g., developing a working therapeutic relationship). This mode of delivering psychological interventions has been found to be feasible and produce clinical outcomes similar to those obtained by traditional face-to-face interventions (Backhaus et al., 2012). In the case of adolescent SAD, it may have additional advantages. On the one hand, it may be aligned with the adolescents’ overall proficiency and availability to use these tools in the service of their mental health (King et al., 2006). On the other hand, it may allow for higher accessibility of the intervention for those experiencing SAD, who are often reluctant to seek specialized help (McDonagh et al., 2022). To counter this reluctancy, this research project worked with a non-treatment seeking sample using a restrictive (limited to well-established SAD diagnosis) and inclusive (included adolescents with any comorbidities) recruitment process. This process, combined with descriptive values on the self- and therapist reported change being very similar between our sample and those reported in previous works, lead us to believe that the current sample is representative of the usual experiences of SAD in adolescence.

Still, current findings should be considered prudently, due to the following limitations. Though this research project included a wait-list control group, participants in that group were not asked to weekly report on their social anxiety symptoms or how they had been changing; likewise, no therapist weekly evaluated their symptoms or change thereof. As such, we are not able to compare that control group with the current intervention group concerning change throughout treatment, unlike what was done in this respect by Leigh and Clark (2022). Such comparison might be accomplished in the future by using a psychological placebo intervention, which would allow for a more conclusive understanding of the benefits of CT on the naturally unremitting course of SAD (Goldin et al., 2014; Stein et al., 2017) and/or in comparison with alternative intervention conditions (e.g., attention control as used by Warner et al., 2007). The current work also does not report data on whether participants fulfilled criteria for SAD by post-intervention, given that such assessment was planned only for after the second follow-up moment (i.e., 6-months after post-intervention). As such, current findings should be interpreted as preliminary indicative of efficacy based only on change during the course of treatment. Additionally, due to the relatively low sample size, our regression models only accounted for the longitudinal data structure by incorporating subject-specific intercept and therefore effectively model population-based trajectories across sessions. Finally, both adolescents and therapists’ reports may have been favorably biased and were dependent on comparing current assessments with a baseline moment that was subjected to recollection bias; concerning therapist, though we suggest one of the therapists may have perceived more evident gains based on their broader previous clinical experience, we did not collect this and other therapist-related variables that could have impacted our outcomes. Though the mean values for both of our measures are close to those reported in other works (Zaider et al., 2003; Hayes et al., 2008) thus allowing for some confidence in the current findings, future works might consider reports that are either blinded or considered in complement to other real-time change indicators (e.g., observational methods), as well as assessing participant- and therapist-related variables that might come to optimize therapeutic outcomes.

The current work presents preliminary evidence, based on change throughout therapy, that the CT@TeenSAD – a remote individual therapy based on CT for adolescents diagnosed with SAD (Leigh and Clark, 2018a,b) –, may be useful in changing the course of adolescent social anxiety. Future works should continue to explore this issue, by using rigorous methodologies (i.e., randomized-controlled trials) that may further sustain the accessibility, efficacy, and effectiveness of this intervention approach. Given that SAD in adolescence is usually a precursor of maladaptive developmental trajectories (Jystad et al., 2021), it seems paramount to devise evidence-based ways that may help adolescence look at social events from a more realistic, flexible, and behaviorally approaching perspective.

## Data availability statement

Datasets will be made available by the corresponding author upon reasonable request.

## Ethics statement

The studies involving human participants were reviewed and approved by the Comissão de Ética e Deontologia da Investigação da Faculdade de Psicologia e Ciências da Educação da Universidade de Coimbra. Written informed consent to participate in this study was provided by the participants’ legal guardian/next of kin.

## Author contributions

PV conceived and designed the study, conducted the final statistical analysis, and wrote the first draft and subsequent reviews of the manuscript. AG-Á organized the database and performed the initial statistical analysis. DF wrote sections of the manuscript. AM designed the data analyses plan and supervised the final statistical analysis and presentation of the results. DR revised and approved the final version of the manuscript. All authors contributed to manuscript revision, read, and approved the submitted version.

## Funding

This research was supported by the FEDER - European Social Fund - through the COMPETE 2020 - Operational Program for Competitiveness and Internationalization (Project reference POCI-01-0145-FEDER-029445), and by Portuguese funds through the Portuguese Foundation for Science and Technology (Project reference PTDC/PSI-ESP/29445/2017 & UIDB/PSI/00730/2020).

## Conflict of interest

The authors declare that the research was conducted in the absence of any commercial or financial relationships that could be construed as a potential conflict of interest.

## Publisher’s note

All claims expressed in this article are solely those of the authors and do not necessarily represent those of their affiliated organizations, or those of the publisher, the editors and the reviewers. Any product that may be evaluated in this article, or claim that may be made by its manufacturer, is not guaranteed or endorsed by the publisher.

## Footnotes

^1^https://clinicaltrials.gov/ct2/show/NCT04979676; further information can be obtained upon reasonable request made to the corresponding author.
