# Supplementary material for: Remotely delivered cognitive therapy for social anxiety disorder in adolescence: Preliminary efficacy evidence based on changes throughout treatment
Source: Front Psychol. 2023 Jan 10;13:915677. doi: 10.3389/fpsyg.2022.915677 (PMC9901541; doi:10.3389/fpsyg.2022.915677)
Supplement: Supplementary file 1 [file Table_1.docx]

Supplementary Material

# Table S1: Structure of the CT@TeenSAD intervention

| Module | Session | Theme | Key message of the session |
| --- | --- | --- | --- |
| 1: How socially anxious minds work according to Cognitive Therapy | 1 | Deriving the cognitive model on Social Anxiety Disorder | Social anxiety arises as an alert for us to try to prevent expected negative outcomes from happening (i.e., practicing safety behaviors and becoming self-focused). |
|  | 2 | The impact of self-focused attention and safety behaviors on social anxiety | Though intending to secure positive social outcomes, self-focused attention and safety behaviors contribute to maintain symptoms of social anxiety. |
|  | 3 | The impact of changing the focus of attention on social anxiety | When focused on the social event, a more realistic perspective on the self and on others’ reaction arises, which is usually not as negative as expected. |
| 2: Developing attention flexibility and management of pre and post-event processing | 4 | Attention flexibility | It is possible to shift attention inwards and outwards and thus discover a richer world outside our scary mind. |
|  | 5 | Pre and post-event planning | The moments before and after an event may be used to plan how to engage with the feared social situation in a productive way. |
| 3: Behavioral Experiments | 6 | Behavioral experiment: performing in social events | Ones’ social performance is not felt or perceived as bad as anticipated if one engages with the social event, by shifting attention externally and dropping safety behaviors. |
|  | 7 | Behavioral experiment: positive interaction in social events | Receiving positive feedback from others may help question our negative expectations about ourselves and others. |
|  | 8 | Behavioral experiment: interaction in ambiguous social events | Expressing my thoughts and opinions allows me to present myself to others in a more interesting way and to establish more positive relationships. |
|  | 9 | Behavioral experiment: mistakes happen | Mistakes are a normal part of social interactions that can and should be seen as a learning opportunity. |
| 4: Reviewing gains and relapse prevention | 10 | Where am I and where am I going from here | Engaging with the outside world and questioning our worst fears are ongoing processes that will gradually and continuously help us get closer to our social goals. |
| Note: For detailed information on the goals and exercises of each session, please contact the corresponding author | | | |

| Table S2: Model formulas used for primary and secondary research for observations from individuals $i=1,\ldots, n$ for session $j=2, \ldots, 10$ adjusted for baseline-severity (${bsl\_severity}_{i}$). Note that the overall random intercept is the sum of the population intercept and the subject specific deviation from it: $\beta_{0}+ \gamma_{0i}$ | |
| --- | --- |
| Primary goal | |
|  | Main effect of session on self-reported change |
|  | $\mathrm{SASCI}_{ij}= \beta_{0}+ \gamma_{0i}+ \beta_{1}\mathrm{session}_{ij} +\beta_{2}{bsl\_severity}_{i}+ \varepsilon_{ij}$ |
|  | Main effect of session on therapist-reported change |
|  | ${CGI\_SA}_{ij}= \beta_{0}+ \gamma_{0i}+ \beta_{1}\mathrm{session}_{ij} +\beta_{2}{bsl\_severity}_{i}+ \varepsilon_{ij}$ |
| Secondary goals | |
|  | Main effect of session and therapist on self-reported change |
|  | $\mathrm{SASCI}_{ij}= \beta_{0}+ \gamma_{0i}+ \beta_{1}\mathrm{session}_{ij} +\beta_{2}{bsl\_severity}_{i}+\beta_{3}\mathrm{therapist}_{i}+ \varepsilon_{ij}$ |
|  | Main effect of session and therapist on therapist-reported change |
|  | ${CGI\_SA}_{ij}= \beta_{0}+ \gamma_{0i}+ \beta_{1}\mathrm{session}_{ij} +\beta_{2}{bsl\_severity}_{i}+\beta_{3}\mathrm{therapist}_{i}+ \varepsilon_{ij}$ |
|  | Main effect of session and participants’ sex on self-reported change |
|  | $\mathrm{SASCI}_{ij}= \beta_{0}+ \gamma_{0i}+ \beta_{1}\mathrm{session}_{ij} +\beta_{2}{bsl\_severity}_{i}+\beta_{3}{s\mathrm{ex}}_{i}+ \varepsilon_{ij}$ |
|  | Main effect of session and participants’ sex on therapist-reported change |
|  | ${CGI\_SA}_{ij}= \beta_{0}+ \gamma_{0i}+ \beta_{1}\mathrm{session}_{ij} +\beta_{2}{bsl\_severity}_{i}+\beta_{3}\mathrm{sex}_{i}+ \varepsilon_{ij}$ |

# Table S3: Means and standard deviations for self-reported and therapist-reported change across intervention sessions

|  | SASCI | | CGI-SA | |
| --- | --- | --- | --- | --- |
|  | Min-Max | Mean (SD) | Min-Max | Mean (SD) |
| Session 2 | 12-23 | 16.04 (2.22) | 4-5 | 4.14 (0.36) |
| Session 3 | 11-17 | 14.71 (1.68) | 3-5 | 4.52 (0.60) |
| Session 4 | 7-16 | 13.43 (2.04) | 4-6 | 4.90 (0.44) |
| Session 5 | 6-17 | 12.98 (2.52) | 4-6 | 5.14 (0.48) |
| Session 6 | 6-15 | 12.57 (2.46) | 5-7 | 5.38 (0.59) |
| Session 7 | 6-15 | 12.07 (2.59) | 5-7 | 5.57 (0.68) |
| Session 8 | 7-14 | 11.05 (2.22) | 4-7 | 5.81 (0.81) |
| Session 9 | 6-14 | 10.69 (2.24) | 5-7 | 5.90 (0.70) |
| Session 10 | 5-13 | 9.33 (2.55) | 5-7 | 6.10 (0.70) |
| Note: SASCI: Social Anxiety Session Change Index; CGI - Clinical Global Impression – Social Anxiety; Min = Minimum value; Max = Maximum value. Only values for sessions 2 through 10 are described because ratings imply a comparison with the beginning of treatment, which was not possible for the first session of the intervention. | | | | |

# Table S4: Planned pairwise comparisons between session for self-reported change (SASCI)

| Session | | Mean difference | p-value | 95% CI | |
| --- | --- | --- | --- | --- | --- |
|  |  |  |  | Lower Bound | Upper Bound |
| 2 | 3 | 1.33 | 0.493 | -0.62 | 3.29 |
|  | 4 | 2.61 | 0.002 | .065 | 4.58 |
|  | 5 | 3.07 | <0.001 | 1.11 | 5.03 |
|  | 6 | 3.48 | <0.001 | 1.51 | 5.44 |
|  | 7 | 3.97 | <0.001 | 2.01 | 5.94 |
|  | 8 | 5 | <0.001 | 3.04 | 6.96 |
|  | 9 | 5.35 | <0.001 | 3.39 | 7.32 |
|  | 10 | 6.71 | <0.001 | 4.75 | 8.68 |

| Table S5: Linear mixed model for session and participants’ sex as fixed-effects explaining self and therapist-reported symptom change while adjusting for baseline severity | | | | | | | | | |
| --- | --- | --- | --- | --- | --- | --- | --- | --- | --- |
|  |  | SASCI | | | | CGI-SA | | | |
| Predictors | | Estimates | *CI* | *p* | *df* | Estimates | *CI* | *p* | *df* |
|  | (Intercept) | 17.18 | 10.22 – 24.14 | < .001 | 183 | 4.01 | 2.37 | < .001 | 183 |
|  | Session | -0.74 | -0.83 – -0.66 | < .001 | 183 | 0.24 | 0.21 – 0.26 | < .001 | 173 |
|  | Baseline severity | -0.36 | -1.87 – 1.14 | 0.636 | 183 | 0.01 | -0.35 – 0.36 | 0.961 | 183 |
|  | Participants’ sex | 0.66 | -1.44 – 2.76 | 0.536 | 183 | 0.03 | -0.46 – 0.53 | 0.891 | 183 |
| Random Effects | |  |  |  |  |  |  |  |  |
|  | *σ^2^* | 2.41 |  |  |  | 0.23 |  |  |  |
|  | *τ_00_* | 3.16_participant_ |  |  |  | 0.16_participant_ |  |  |  |
|  | *ICC* for complete model/ *ICC* for random intercept only | 0.57/ 0.27 |  |  |  | 0.42/ 0.13 |  |  |  |
|  | *N* | 21_participant_ |  |  |  | 21_participant_ |  |  |  |
| Marginal R2/ Conditional R2 | | 0.405/0.742 |  |  |  | 0.488/0.701 |  |  |  |
| Note: SASCI = self-reported symptom change; CGI-SA = therapist-reported symptom change. Observations = 189. | | | | | | | | | |

Table S6: Planned pairwise comparisons between session for therapist-reported change (CGI-SA)

| Session | | Mean difference | p-value | 95% CI | |
| --- | --- | --- | --- | --- | --- |
|  |  |  |  | Lower Bound | Upper Bound |
| 2 | 3 | -0.38 | .357 | -0.90 | 0.14 |
|  | 4 | -0.76 | <.001 | -1.25 | -0.24 |
|  | 5 | -1.00 | <.001 | -1.52 | -.048 |
|  | 6 | -1.24 | <.001 | -1.76 | -0.72 |
|  | 7 | -1.43 | <.001 | -1.95 | -0.91 |
|  | 8 | -1.67 | <.001 | -2.19 | -1.15 |
|  | 9 | -1.76 | <.001 | -2.28 | -1.24 |
|  | 10 | -1.95 | <.001 | -2.48 | -1.43 |
